# A comparative study on nursing students’ attitudes towards death and palliative care before and after their clinical internship in China

**DOI:** 10.1097/MD.0000000000047211

**Published:** 2026-01-23

**Authors:** Qingmei Ju, Yang Liu, Kim Lam Soh, Yiqiang Guo

**Affiliations:** a Department of Pediatrics, Emergency General Hospital, Beijing, China; b Nursing Department, School of Medicine, Xia Men University, Xiamen, Fujian Province, China; c Department of Nursing, Universiti Putra Malaysia, Selangor, Serdang, Malaysia; d Teaching Department, Kiang Wu Nursing College of Macau, Macau, China.

**Keywords:** attitudes towards death, care attitudes, death education, nursing students, palliative care

## Abstract

**Background:**

Nurses play a central role in delivering palliative care (PC), and their attitudes toward death directly affect the quality of care provided. Clinical internships represent a critical transition for nursing students, bridging theoretical knowledge and practical experience, and shaping their development as professional caregivers. However, research in this area remains limited. This study aims to understand the changes in attitudes towards death and PC among nursing undergraduate students before and after clinical internships in China.

**Methods:**

A longitudinal survey was conducted with 224 nursing undergraduate students from a medical college selected as the study participants. The Chinese version of the Death Attitude Profile-Revised, Frommelt Attitude Toward Care of the Dying Scale, and the Palliative Care Competence Self-Assessment Questionnaire were used for the survey.

**Results:**

After the internship, nursing students’ scores for death fear, death anxiety, avoidance acceptance, and approach acceptance attitudes were higher than before the internship; the score for natural acceptance attitude was lower than before the internship. The total score of the PC competence after the internship (100.60 ± 9.80) was lower than before the internship (103.74 ± 7.75), and the differences were statistically significant. The self-assessed competence percentages for various items of PC ranged from 31.8% to 50.5%, all lower than before the internship.

**Conclusion:**

Clinical practice poses real challenges to nursing students, resulting in a decline in positive attitudes towards death and PC competence. It is necessary to optimize clinical teaching on PC, address existing deficiencies, enhance nursing students’ positive attitudes towards death and PC competence, and ensure the adequacy and stability of the PC workforce.

## 1. Introduction

Palliative care (PC) represents a vital component of healthcare, offering a holistic approach to support individuals of all ages facing serious illnesses and associated suffering, especially those approaching the end of life.^[1]^ The main content includes pain and other symptom control, comfort care, psychological, spiritual and social support, are directly related to the “quality of death” of end-stage patients.^[2]^ Although palliative care can improve the quality of life of patients and families, it remains underutilized.^[3]^ There are various factors contributing to the limited universal access to palliative care, including disparities in wealth across different regions, local laws and cultural attitudes, as well as discrimination against minority groups.^[4]^ A common cultural factor across nearly all countries, including the most developed Western nations, is a widespread lack of awareness about palliative care.^[5]^ Medical workers around the world have recognized the importance of palliative care education, but the palliative care education for medical staff is not enough.^[6]^ As noted in the literature,^[7]^ research examining student nurses’ knowledge of palliative care in the Middle East is significantly limited, particularly concerning their attitudes. Nurses exhibited favorable perceptions of organizational and resource-related aspects of palliative care, yet showed elevated moral and ethical concerns within the clinical subdimension.^[8]^

Clinical training is crucial in this regard, as it allows nursing students to apply their theoretical knowledge and develop the practical skills needed to navigate such emotional and ethical challenges in real healthcare environments.^[9]^ Through clinical experiences, nursing students enhance their professionalism by gaining a deeper understanding of and firsthand experience in providing care. These experiences are essential in helping students build the skills needed for their nursing careers after graduation, offering a solid foundation that prepares them to navigate various healthcare settings and situations they may encounter in the future. However, the literature^[10]^ has underscored the scarcity of research exploring when and how palliative care principles and practices are introduced within undergraduate nursing curricula, indicating a critical gap in the timing and integration of such content in nurse education. A survey conducted across 91 nursing schools found that many graduates feel inadequately prepared to handle the complexities of death and dying, highlighting a gap in their clinical training and emotional readiness.^[11]^

Death education offers a valuable opportunity for future healthcare professionals to address the essential aspects of palliative care and develop cognitive skills that enhance their ability to communicate and build relationships. Through this education, they gain an understanding of the various ways death can manifest and the different cultural and personal meanings associated with it.^[12,13]^ Chinese nurses encounter challenges in their clinical skills due to insufficient palliative care education during their nursing studies,^[14]^ as well as a lack of specialized training at their workplaces.^[15,16]^ The negative attitudes were manifested through feelings of fear when confronted with death and a reluctance to think about or discuss death-related topics.^[17]^ Nurses make up the largest segment of the healthcare team responsible for caring for terminally ill patients,^[18]^ and they have a vital role in preserving the dignity, self-respect, sense of worth, and overall well-being of these patients through their interactions.^[19]^ It is essential for nurses in various clinical environments to receive training on how to address the needs of patients nearing the end of life.

There is an urgent need for a comprehensive palliative care training system in China, and assessing the effectiveness of this system will provide credible evidence and high-quality scientific data to drive progress.^[20]^ Therefore, the purpose of this study is to investigate the status of death attitudes, palliative care attitudes and competencies of nursing students before and after practice, and to provide preliminary data for future palliative care education for nursing students.

## 2. Materials and methods

### 2.1. Design and sample

#### 2.1.1. General information

A convenience sampling method was employed to conduct a clustered questionnaire survey among undergraduate nursing students at a medical college. Inclusion criteria were as follows: undergraduate students; majoring in nursing; and completed 10 months of clinical internship. Prior to the internship, a total of 230 questionnaires were distributed to nursing students, of which 224 were completed and returned, yielding a response rate of 97.4%. Following the completion of the internship, the same number of questionnaires were distributed again, with 216 valid responses collected, corresponding to a response rate of 93.9%.

#### 2.1.2. Questionnaire survey

The questionnaire consists of 3 parts, including: the death attitude profile developed by Wong et al^[21]^ in 1994, which includes 5 dimensions: fear of death, death avoidance, neutral acceptance, approach acceptance and escape acceptance, with a total of 32 items. Domestic researchers conducted cultural adjustments on the scale to form a Chinese version suitable for domestic use. The higher the score of each dimension, the more the attitude tends to the death attitude of this dimension. In this study, the Cronbach’s α coefficient was 0.839, indicating good reliability and validity. The Frommelt Attitude Toward Care of the Dying Scale was developed by Dr Frommelt of the United States^[22]^ to measure nurses’ attitudes towards end-of-life care. It includes 30 items. Dr Wang Liping adapted this scale to form the Chinese version of Frommelt Attitude Toward Care of the Dying Scale-B, which consists of 29 items and has good reliability and validity.^[23]^ The Cronbach’s α coefficient in this study was 0.742; this scale has 6 dimensions and is scored according to the Likert 5-point method (strongly disagree, disagree, uncertain, agree, strongly agree), with the total score ranging from 29 to 145 points. A higher score indicates a more positive attitude towards care. A self-developed palliative care competence self-assessment questionnaire, which includes 7 items. The specific items are detailed in Table 3.

### 2.2. Quality control

The questionnaire survey was conducted by trained investigators, who explained the purpose and precautions of the survey before distribution, and the participants voluntarily completed it. The questionnaires were collected on the spot and any omissions or ambiguities found were immediately corrected.

### 2.3. Data analysis

Using IBM SPSS 26.0 software (IBM Corp., Armonk) to describe the general demographic characteristics of the participants and the current state of palliative care competence; to compare death attitudes and palliative care attitudes before and after the nursing students’ internship, and to perform self-assessment of palliative care competence, using a *t*-test or chi-square test, with *P* < .05 indicating statistical significance.

### 2.4. Ethics statement

The study protocol was approved by the Institutional Review Board of Xiamen University, China (No. XDYX2021027).

## 3. Results

### 3.1. Basic demographics of nursing students post-internship

About 204 (94.44%) of nursing students after internship are girls, 160 (74.07%) are from rural areas, 82 (37.96%) avoid talking about death, and 194 (89.81%) grow up in nuclear families, 10 (4.63%) were from direct families, and 12 (5.56%) were from single-parent families. About 114 people (52.78%) have taken care of dying patients in practice, and 76 people (35.19%) have received death education or palliative care education in practice, the duration ranges from 1 to 5 hours.

### 3.2. Comparative analysis of death attitudes pre- and post-internship

Nursing students had higher scores in the 4 dimensions of fear of death, death avoidance, approaching acceptance, and escape acceptance after the practice; the scores of the neutral acceptance dimension were lower than those before the practice, and the differences were statistically significant (see Table 1).

**Table 1:** Comparative analysis of death attitudes of nursing students pre- and post-internship.

Time	Fear of death	Death avoidance	Neutral acceptance	Approach acceptance	Escape acceptance
Pre-	2.93 ± 0.62	2.96 ± 0.75	4.00 ± 0.51	2.76 ± 0.56	2.56 ± 0.72
Post-	3.26 ± 0.46	3.33 ± 0.71	3.78 ± 0.58	3.03 ± 0.46	2.78 ± 0.66
*t*	8.86	7.58	−6.00	7.81	4.76
*P*	<.001	<.001	<.001	<.001	<.001

### 3.3. Comparative analysis of attitudes towards palliative care pre- and post-internship

The total score for attitudes towards palliative care prior to the nursing students’ internship was 103.74 ± 7.75, and post-internship it was 100.60 ± 9.80. Statistical testing revealed no significant difference. After dividing the scores of each dimension of attitudes towards palliative care post-internship by the number of items, the score for “attitude towards the interests of end-of-life care patients” was 3.70, “attitude towards caring for end-of-life patients” was 3.40, “attitude towards the necessity of family support” was 3.94, and “attitude towards caring for patients’ families” was 3.87. The lowest score was for “attitude towards communication with end-of-life care patients” (2.65), followed by “attitude towards addressing the anxiety and uneasiness of caring for end-of-life patients” (3.17). The scores for each dimension are provided in Table 2.

**Table 2:** Comparison of attitudes towards palliative care of nursing students pre- and post-internship.

	$\bar{x}$ ± SD	*t*	*P*
Total score of attitudes toward palliative care		3.661	<.001
Pre-	103.74 ± 7.75		
Post-	100.60 ± 9.80		
Attitudes towards the well-being of terminally ill patients (6 items)		1.292	.197
Pre-	22.51 ± 2.40		
Post-	22.17 ± 3.07		
Attitudes towards caring for terminally ill patients (6 items)		2.341	.020
Pre-	21.10 ± 2.80		
Post-	20.42 ± 3.28		
Attitudes towards the necessity of supporting family members (5 items)		2.974	.003
Pre-	20.39 ± 2.26		
Post-	19.71 ± 2.45		
Attitudes towards communication with terminally ill patients (5 items)		−0.152	.884
Pre-	13.23 ± 2.18		
Post-	13.27 ± 2.38		
Attitudes towards caring for patients’ family members (4 items)		4.711	<.001
Pre-	16.46 ± 1.96		
Post-	15.51 ± 2.24		
Attitudes towards addressing the psychological anxiety and unease when caring for terminally ill patients (3 items)		1.803	<.001
Pre-	10.00 ± 1.92		
Post-	9.50 ± 3.53		

SD = standard deviation.

**Table 3:** Comparison of self-assessed competence pre- and post-internship.

Item	Yes (%)	No (%)	χ^2^	*P*
I am able to help manage pain symptoms in terminally ill patients			46.213	<.001
Pre-	142 (67.9)	82 (35.5)		
Post-	67 (32.1)	149 (64.5)		
I am able to assist in managing respiratory symptoms (such as coughing, difficulty breathing) in terminally ill patients			66.151	<.001
Pre-	170 (67.7)	54 (28.6)		
Post-	81 (32.3)	135 (71.4)		
I am able to assist in managing gastrointestinal symptoms (such as nausea, vomiting, constipation, bowel obstruction) in terminally ill patients			59.532	<.001
Pre-	159 (68.2)	65 (31.4)		
Post-	74 (31.8)	142 (68.6)		
I am able to assist in managing neurological/psychological symptoms (such as delirium, seizures, anxiety) in terminally ill patients			41.932	<.001
Pre-	155 (65.1)	69 (34.2)		
Post-	83 (34.9)	133 (65.8)		
I can identify the emotional needs of terminally ill patients and their families			16.903	<.001
Pre-	131 (60.9)	93 (41.3)		
Post-	84 (39.1)	132 (58.7)		
I can provide bereavement care for terminally ill patients and their families			0.267	.605
Pre-	91 (49.5)	133 (52.0)		
Post-	93 (50.5)	123 (48.0)		
I can identify the spiritual needs of terminally ill patients and their families			11.721	.001
Pre-	133 (58.8)	91 (42.5)		
Post-	93 (41.2)	123 (57.5)		

### 3.4. Self-assessed competence in palliative care among nursing students pre- and post-internship

Prior to the internship, the percentage of nursing students who self-assessed their ability to help manage symptoms in terminally ill patients ranged from 49.5% to 68.2%. Post-internship, this range was 31.8% to 50.5%. The difference was statistically significant, as detailed in Table 3.

## 4. Discussion

This study revealed that the palliative care attitude scores of nursing students decreased after the internship compared to before. In line with the Lancet Commission on the value of death^[24]^ and the perspectives already highlighted in existing literature,^[25]^ it is feasible to reintroduce the topics of death and dying into people’s everyday lives, including the lives of students. Therefore, equipping nursing students with the skills to care for dying patients is a crucial component of their professional training, the need for relevant intervention education during nursing students’ internships.^[20,26]^

### 4.1. Enriching teaching mode of death education

The scores of nursing students’ “natural acceptance” of death attitude were the highest before and after the internship, which is consistent with the survey results of^[27]^ on college students’ death attitudes, indicating that nursing students tend to believe that death is a part of life, and life and death are a natural process. However, the attitude of nursing students towards accepting death naturally fades after the internship, and the scores of fear of death, death avoidance, approach acceptance and escape acceptance increase compared with before the internship. Students who received a theoretical education on providing care for terminal patients have positive attitudes towards the principles of a good death and spiritual care.^[28]^ Death education has a positive effect on the positive cultivation of the nursing students’ death attitude. In this study, 76 people (35.2%) have received death education or palliative care education during their internship, with a duration of 1 to 5 class hours. It demonstrated that the current clinical teaching lacks death education, and it is difficult for nursing students to internalize positive concepts of life and death. Nursing students’ attitude towards death is mainly evasion, and death education has a positive effect on the positive cultivation of the death attitude of the research subjects.^[20]^ The training rate of nurses receiving death education is only 29.1% in China.^[29]^ It is suggested that the normalization of death is an important part of death education. Enriching the teaching mode of death education is the key content of palliative care education. Education should involve the 2-way perspective of doctors and patients, encourage students to reflect on their own death attitudes through education and anxiety, fear and other emotions, thereby affecting the attitude of nursing students in palliative care. Clinical teaching carries out death education at different stages to improve the ability of nursing students to provide emotional support for patients and their families during palliative care,^[30]^ so that nursing students can maintain continuous learning opportunities and motivation, supported by appropriate guidance and a wealth of knowledge, helps them build confidence in caring for terminally ill patients. This enables them to view end-of-life care as a rewarding and fulfilling experience.^[31]^

### 4.2. Self-efficacy improves palliative care attitude

After the internship, the attitude scores of the nursing students for palliative care decreased compared with those before the internship, and the difference was statistically significant. Nursing students recognize the importance of palliative care, feel confident in addressing patients’ terminal symptoms, and can provide emotional support to patients or family members when they are depressed. They also discuss the process of death with patients. However, occasional psychological shock and discomfort can reduce their enthusiasm for end-of-life care, the absence of structured support, debriefing, or culturally sensitive supervision during clinical practice may further aggravate students’ stress and reduce their self-efficacy in palliative care delivery.^[32,33]^ Before and after the practice, the scores of “attitude of communicating with terminal patients” and “attitudes toward anxiety and unease when caring for terminal patients” were low. Palliative care starts with having honest conversations, especially when aggressive treatments might cause more harm than beneficial.^[34]^ Having open communication about illness and death between patients and their family members can improve the well-being of caregivers. Research has shown that such communication is linked to lower caregiver stress^[35]^ and greater emotional relief.^[36]^ A lack of proper palliative care training for nurses caring for terminally ill or dying patients can lead to poor communication, a lack of empathy, and even avoidance of the patient, all of which can increase anxiety for both the patient and their family.^[37]^ Self-efficacy is positively correlated with care attitudes,^[38]^ and self-efficacy plays a key role in the process of caring for terminally ill patients.^[39]^ Low self-efficacy will cause nursing students to avoid providing nursing services for patients, resulting in adverse outcomes for patients and prone to job burnout.^[40]^ The research of Moreland^[41]^ shows that the simulated nursing care carried out by palliative care education can improve the self-efficacy and nursing attitude of nursing students, so it is necessary to improve the nursing attitude by improving the self-efficacy of intern nursing students.

After a 10-month internship, the nursing students’ self-assessment of palliative care competency decreased. Internship experiences may expose students to high moral and ethical distress, especially in the absence of proper mentorship and structured learning opportunities, potentially leading to a decrease in confidence and perceived competence. The reviewed literature^[42,43]^ points to high levels of moral/ethical conflict in clinical subdimensions related to palliative care, which could explain why students feel less prepared after real-world encounters. This is compounded by cultural barriers and emotional unpreparedness, particularly in cultures where death is taboo, such as in Confucian-influenced contexts, which may lead students to withdraw from end-of-life care situations rather than engage fully.^[44]^ When facing patients and dealing with actual clinical problems, nursing students may realize the heavy responsibility of life and death, and deeply feel that their knowledge is not enough to help patients and their families cope with the changes brought about by the disease. It also shows that the quality and structure of palliative care education remain inadequate within undergraduate nursing curricula. Specifically, nursing students often lack adequate theoretical grounding and practical training in core palliative care competencies before entering clinical settings.^[45]^ Moreover, the palliative care education received by nursing students during the internship is not deep enough, the clinical teaching process does not pay enough attention to palliative care, and the clinical teaching system of palliative care is not sound enough. We can learn from Liu Xiangyu’s^[46]^ palliative care specialist nurse training system based on Bloom’s goal teaching theory, guide clinical teaching from the 3 frameworks of cognitive domain, emotional domain, and operation skill domain, and promote nursing students to have higher professional identity and job satisfaction.

## 5. Conclusions

There remains a significant gap between the supply and demand of palliative care services. Clinical internships serve as a crucial transition period for nursing students, moving from theory to practice, and from student to professional caregiver. Effective clinical teaching is essential in this process. It’s necessary to enhance the clinical training management system and address existing shortcomings to ensure a sufficient and stable workforce in palliative care. This will equip nursing students to meet the current health needs of the Chinese population in their future work, expedite the development of a standardized palliative care service system in China, and effectively foster the comprehensive growth of palliative care services.

### 5.1. Limitations

This study has several limitations that should be acknowledged. First, the sample was limited to nursing students from a single institution, which may affect the generalizability of the findings. Second, the reliance on self-reported data may introduce response bias, as students’ perceptions of their competence might not fully reflect their actual skills. Third, the cross-sectional design limits the ability to establish causality between internship experience and changes in palliative care competence. Future research with larger, more diverse samples and longitudinal designs would provide more robust evidence.

## Acknowledgments

The authors express their gratitude to all the participants who participated in this study.

## Author contributions

**Conceptualization:** Qingmei Ju, Yiqiang Guo.

**Data curation:** Qingmei Ju, Yang Liu.

**Methodology:** Qingmei Ju, Kim Lam Soh.

**Writing – original draft:** Qingmei Ju, Yiqiang Guo.

**Writing – review & editing:** Qingmei Ju, Kim Lam Soh, Yiqiang Guo.
